# Access to finance from different finance provider types: Farmer knowledge of the requirements

**DOI:** 10.1371/journal.pone.0179285

**Published:** 2017-09-06

**Authors:** Eliana Wulandari, Miranda P. M. Meuwissen, Maman H. Karmana, Alfons G. J. M. Oude Lansink

**Affiliations:** 1 Business Economics, Wageningen University, Wageningen, The Netherlands; 2 Faculty of Agriculture, University of Padjadjaran, West Java, Indonesia; Universidad de Piura, PERU

## Abstract

Analysing farmer knowledge of the requirements of finance providers can provide valuable insights to policy makers about ways to improve farmers’ access to finance. This study compares farmer knowledge of the requirements to obtain finance with the actual requirements set by different finance provider types, and investigates the relation between demographic and socioeconomic factors and farmer knowledge of finance requirements. We use a structured questionnaire to collect data from a sample of finance providers and farmers in Java Island, Indonesia. We find that the most important requirements to acquire finance vary among different finance provider types. We also find that farmers generally have little knowledge of the requirements, which are important to each type of finance provider. Awareness campaigns are needed to increase farmer knowledge of the diversity of requirements among the finance provider types.

## Introduction

Availability of finance is important for sustaining the production of agricultural commodities [1, 2]. Moreover, availability of finance contributes to increased income [3, 4], productivity improvements [5, 6] and efficiency improvements [7]. Previous studies have identified the different types of finance providers [8–10]. Lack of access to finance is a major problem for the rural poor, especially in developing countries [11, 12].

A number of studies have shown the importance of the knowledge of finance providers. Swinnen and Gow [13] showed that access to finance might be constrained because finance providers lack information about the agricultural sector. Knowledge-based factors influenced the assessment of the credit-worthiness of borrowers [14] and the credit-rating of applicants [15]. Armendariz and Morduch [16] pointed out that financial services were improved by lenders’ information about borrowers.

Financial knowledge is important for financial decision making [17]. A lack of financial knowledge, especially of farmers, can explain the low income of farmers and slow economic growth [18]. Despite the importance of financial knowledge, some studies have explored the financial knowledge of farmers [18–21], but none have studied farmer knowledge of the requirements to obtain finance from different finance provider types. Analysing farmer knowledge of the requirements of finance providers can help identify opportunities to improve farmers’ access to finance. In this context, the first objective of this study is to compare farmer knowledge of the finance requirements of different finance provider types with the requirements set by each finance provider type. The second objective is to investigate the relation between demographic and socioeconomic factors and farmer knowledge of finance requirements. The results of this study provide insights, which are useful to design strategies to improve farmer knowledge of finance and subsequent access to finance.

The empirical application focuses on a sample of horticultural farmers in Indonesia. Agriculture is an important and growing sector in the Indonesian economy, accounting for 14.7% of Indonesian gross domestic product in 2011 [22]. The export value of horticulture grew 22.8% per year from 2007 until 2011 [22]. Horticulture can become an important source of income for Indonesian farmers as it has high economic value.

## Finance from different types of finance providers in Indonesia

The Indonesian government aims to develop horticulture, among other agricultural sectors, by improving access to credit [23]. Farmers can access finance from various finance providers, such as banks, credit associations, private moneylenders, relatives [9], micro finance institutions (MFI) [8], traders [24] and credit cooperatives [25]. This section discusses in detail the requirements to obtain finance, which are set by the different finance provider types in Indonesia.

### Finance from banks

Farmers can access finance from banks by applying for commercial credit. The loan duration depends on the policy of the bank, the type of commercial credit and the amount of credit applied for. For instance, farmers may apply for working capital credit for a duration of 12 or 36 months, dependent on the amount of credit needed. A farmer can apply for commercial credit by completing a credit application form and providing supporting documents. The supporting documents are required to prove identity and provide evidence of collateral. Examples of supporting documents include: photocopies of the identity cards of the applicant and his/her partner in case of marriage, and photocopies of land certificates, salary slips, deposits or a certificate of vehicle ownership. Banks then analyse the credit application by interviewing the applicant or conducting a farm visit.

Farmers can also apply for a subsidised credit programme, offered by banks collaborating with the Indonesian government. An example is the programme of micro credit loans (*KUR*-*Kredit Usaha Rakyat*). The KUR aims to help farmers, especially those who do not have any collateral. The KUR guidelines of the Agricultural Ministry of Indonesia [26] specify a number of conditions, such as the size and length of the loan, which depend on the type of the KUR. For micro KUR, for instance, farmers may borrow up to IDR 20 million (the exchange rate in 2013, US$1 = IDR 10,445) for a maximum of 3 years. To obtain credit from the KUR, an applicant needs to make a proposal, which is endorsed by the local agricultural officer. Next, a form for credit application is completed and submitted together with the proposal to a selected bank. The bank analyses the feasibility of the proposal and conducts a farm visit to decide on approval. The KUR programme is run by a selected group of banks.

### Finance from micro finance institutions (MFI)

MFI play an important role in the provision of credit to farmers in Indonesia. To obtain credit from MFI, farmers are required to register as a member of the MFI. Following membership, a farmer is allowed to submit a credit application. The financing from MFI benefits small farmers who cannot obtain credit from banks.

### Finance from farmers’ associations

Farmers’ associations generally exploit many activities, including selling the products of their members. Farmers’ associations have access to finance, especially through government programmes. Farmers’ associations channel government aids to farmers who are members of the farmers’ association. The government provides the associations with cash aids, seed and fertilizer aids, farm equipment and subsidies to purchase seed and fertilizer. Cash aids are provided through the rural agribusiness development programme (*PUAP*-*Pengembangan Usaha Agribisnis Perdesaan*). This programme targets farmers’ associations to motivate farmers to increase production. Governmental provision of finance via farmers’ associations is based on past experience in Indonesia, showing that a group approach strongly affects the success of a programme [27]. To access this source of finance, farmers’ associations need to submit a proposal of their agricultural business plan to the local government. The local government verifies the proposal and documents. Evaluation criteria are location in a PUAP area, sufficient human resources for operating agribusiness activities and active farm management by farmers [23]. If the criteria are fulfilled, the proposal is submitted to the director of finance at the Agricultural Ministry of Indonesia. The associations then distribute the finance obtained from the government to their members. Finance is provided in-kind, i.e. by distributing agricultural inputs to their members. In return, some farmers’ associations require members to sell their products to the farmers’ association under the conditions set in the contract between association and members.

### Finance from traders

Some traders in Indonesia provide finance in-kind to those farmers who have contracts with the traders. The contract is set by the trader and farmers together and stipulates rules for production, such as cultivation procedures, the amount of produce to be delivered and prices. Both traders and farmers benefit from this contract, as farmers have both a buyer for their products and a guaranteed price, and traders have guaranteed continuity of supply.

### Finance from agricultural input kiosks

Some agricultural input kiosks provide finance to farmers by selling inputs, such as seed, fertilizer and pesticides, for a flexible payment. The flexible payment means that farmers can pay for the input a week to a month after purchasing. Some kiosks allow farmers to pay for the inputs after harvesting, as long as the farmers agree to sell the harvest to the kiosk.

### Finance from other finance providers

Farmers can also obtain finance from other finance providers, such as family, relatives and neighbours. Informal finance is a major source of finance, especially for poor people [10]. Interest is not usually charged for informal finance [28].

## Research methods

This section describes the conceptual framework, procedure of survey, the survey of finance providers and farmers, and the analytical methods used in this study.

### Conceptual framework

The conceptual framework is presented in S1 Fig. The finance requirements are based on the theory of risk assessment in finance, reported by Miller [29]. The determinants of farmer knowledge are derived from a literature review. The factors that influence the provision of finance are presented on the left hand side of S1 Fig. Following Abbadi and Abu Karsh [30] we assume that finance providers focus on the five Cs of credit worthiness i.e. collateral, character, capacity, capital and condition. In this study, we use the following definitions for the five Cs. *Collateral* is defined as a farmer’s guarantee letters, such as land and vehicle certificates. *Character* is defined as a farmer’s history of loan repayments. *Capacity* is defined as the profitability of farms. *Capital* is defined as savings and *condition* as the national political and macroeconomic situation in the country, which influences the decisions of finance providers on the distribution of finance. Relevant factors that affect the national situation include inflation and national elections.

We included additional variables in the finance provision part of the conceptual framework based on a review of relevant literature. Previous studies have found that access to finance is determined by the amount of credit applied for [15], the ability of farmers to manage their farms as shown by their experience [31–32], farm size [31–34], and membership of a village or farmers’ association [35]. Furthermore, Glover [36] pointed out that having a contract in agriculture is not only beneficial for market access, but also eases access to credit. The additional variables included in the finance provision part of our conceptual framework are: *loan size*, defined as the amount of money needed by the farmers; *farmer ability*, defined as the ability of the farmer to manage their farm; *farm size*; *spouse knowledge*, defined as whether the spouse knows about the finance application; *membership* of a registered farmers’ association and presence of a *sales contract*. Some literatures have shown that access to finance is associated by gender [9, 35]. However, we do not use a gender variable in the analysis because we only have very few female respondents (4 out of 434), which is too small to make statistical inference. Informal rules such as social relationships were not considered as a study of Supriatna [37] showed that these are mainly important for informal financial services, i.e. services not covered in our study.

The determinants of farmer knowledge of finance requirements are shown on the right hand side of S1 Fig. Several studies have shown that farmer knowledge is influenced by demographic and socioeconomic factors. Education, farm size, farming experience and finance experience were significant variables that determined farmer knowledge of the risks and benefits of herbicide-tolerant canola in Western Canada [38]. Similarly, Kumar Jha [39] reported that education and size of land holding were important in determining the level of knowledge of social forestry practices. Furthermore, Harrison [40] found that age significantly influenced the knowledge of farmers about financial management practices.

### Procedure of survey

This study is part of a comprehensive project, reviewed and approved by the Assessment Committee of Wageningen School of Social Sciences (WASS). A letter of the project team, accompanied with a letter from the embassy of Indonesia in The Netherlands as well as a permission letter from Faculty of Agriculture, Padjadjaran University, Indonesia were used as supporting letters sent to finance providers. The same letters were used during the farmers’ survey. From the agricultural offices in study areas, verbal permission was obtained. Participation was strictly voluntary and written and oral consent were obtained from finance providers and farmers respectively. With regard to the latter we obtained oral consent because part of the target population had little schooling. The same procedure was followed by among others Reyes-Garcia et al. [41]. Next, participants were introduced to the study purpose and contents before entering the survey. The questionnaires for finance providers (S1 File) and farmers (S2 File) include personal information of participants. Therefore, anonymity was acknowledged and assured with an anonymous data set.

### Survey of finance providers

#### Questionnaire design

We designed the structured questionnaire for the finance providers to identify the most important requirements for farmers to obtain access to finance. We used a five-point Likert-scale (ranking from 1 to 5) to elicit finance providers’ perceptions of the important requirements, following the method of Wyatt and Meyers [42]. We infer the finance requirements from the perceptions of the finance provider representative because we do not know the banks formal evaluation procedures, as these are internal to the finance providers. The questionnaire was pretested to see if we had missed any important requirements.

#### Data collection

During the period from August 2013 to July 2014, we collected data from 43 finance providers, which consist of 5 types of finance providers: 1) banks (6), 2) MFI (5), 3) farmers’ associations (13), 4) traders (6), and 5) agricultural input kiosks (13). These providers all had horticultural farms in their portfolio and the majority of their business in Java, the same region as the surveyed farmers. Contact details for banks and traders were provided by the Agricultural Ministry of Indonesia. Contact details for MFI, farmers’ associations and agricultural input kiosks were obtained from personal contacts, including agricultural officers and agricultural networks. In total, 80 finance providers were contacted, of which 43 agreed to participate in this study.

The 6 banks operate as saving and lending institutions. The banks had 4,800 to 26,000 employees and 24 to 100 years of experience in financing farmers. We did not distinguish between commercial and subsidised credit in the questions on the finance requirements of banks. The 5 MFI had 5 to 19 years of experience in financing farmers. The 13 farmers’ associations had 13 to 248 members and 2 to 40 years of experience. The 6 traders are mainly export traders. These traders had 11 to 23 years of experience providing finance to farmers and had 7 to 460 employees. The 13 agricultural input kiosks had 3 to 16 years of experience providing inputs to farmers. The kiosks had 1 to 4 employees and 30 to 300 farmers as customers. Informal finance providers, such as family, relatives and neighbours, were not included in the survey.

### Survey of farmers

#### Questionnaire design

We designed the questionnaire for farmers to investigate farmer knowledge of the requirements set by different finance provider types. We used a five-point Likert-scale (ranking from 1 to 5) to elicit farmers’ perceptions of the importance of the different finance requirements for each type of provider. The questionnaire was pretested to evaluate consistency and clarity, and to avoid duplicate questions. The questions covered three main areas: demographic variables, socioeconomic variables and knowledge of important requirements to obtain finance. The requirements to obtain finance were identical to those in the questionnaire for the finance providers.

#### Data collection

The survey was conducted from January 2014 to July 2014. The study sites were characterised by mixed commodity farming, as most farmers in Indonesia grow more than one commodity on their farms. We collected data from farmers who cultivate one or more of the following as their main crop: mango, mangosteen, chili and red onion. We selected these four crops because the Indonesian Ministry of Agriculture has identified them as key products for horticultural development in Indonesia [23]. Although mango and mangosteen are perennial crops with potentially different risks and farm assets, survey questions were kept identical across crops.

We selected the study sites based on the value of production for the four crops. According to data from the Central Bureau of Statistics in Indonesia (BPS) [43], Java Island has the highest production of all selected commodities. Therefore, we conducted our survey on Java Island. Java Island is divided into provinces, which are formed by several districts. Two provinces were selected as study sites for each commodity, based on the largest area of production and the potential to develop the selected commodity. We chose the provinces according to data from the BPS [43]: West Java and East Java for mango, and Central Java and West Java for chili, red onion and mangosteen. Within each province, we selected the districts with the highest production during the last five years, again using data from the BPS [43]. In West Java, the chosen districts are: Garut, Tasikmalaya and Ciamis for chili; West Bandung and Majalengka for red onion; Tasikmalaya and Subang for mangosteen; and Cirebon and Indramayu for mango. In Central Java the chosen districts are: Brebes for red onion, Purworejo for mangosteen, and Pemalang and Purbalingga for chili. Finally, we selected Probolinggo and Pasuruan for the production of mango in East Java. The selected provinces and districts are similar in terms of government regulation, infrastructure and market structure.

With regard to the selection of farmers per district, farmers were randomly selected based on farm address data obtained from agricultural officers and personal contacts. After careful selection, our sample contains 434 farmers: 101 producing mango, 103 producing mangosteen, 123 producing chili and 107 producing red onion. The characteristics of the farms in the sample are presented in Table 1.

**Table 1:** Demographic and socioeconomic characteristics of farms in the sample.

Dimension	Variable	Mango (n = 101)	Mangosteen (n = 103)	Chili (n = 123)	Red onion (n = 107)	Overall (n = 434)
Farmer	Age ^a^ (years)	46(0.93)	53(1.11)	41(0.86)	49(0.91)	47(0.52)
	Education level ^a^(years)	9(0.25)	8(0.26)	8(0.25)	7(0.22)	8(0.13)
	Farming experience ^a^ (years)	23(1.13)	31(1.42)	15(0.86)	28(1.17)	24(0.64)
Farm	Farm size ^a^ (hectares)	1.30(0.30)	0.87(0.07)	1.20 (0.20)	0.69(0.15)	1.02 (0.10)
Financeexperience ^b^	Bank (%)	25	25	18	21	22
	MFI (%)	5	17	4	10	9
	Farmers’ association (%)	26	45	63	34	43
	Trader (%)	10	10	46	11	20
	Agricultural input kiosk (%)	8	1	27	47	21

^a^ Mean values with standard errors in parentheses.

^b^ Percentage of respondents who have previously obtained finance from the type of finance provider.

Source: Authors’ calculation

As shown in Table 1, on average, farmers were 47 years old and had 8 years of formal education background. Farmers had an average of 24 years of experience in farming activities. The average farm size was 1.02 hectares, and ranged from 0.69 to 1.30 hectares across the four types of farmers. The highest percentage of finance was obtained from farmers’ associations. Most of the mango, mangosteen and chili farmers had previously obtained finance from farmers’ associations, whereas almost half of red onion farmers had previously obtained finance from agricultural input kiosks.

### Analytical methods

We first determined which requirements are perceived to be the most important to obtain finance from different finance provider types, from the perspective of both finance providers and farmers. The most important requirements were defined as the requirements which scored either 4 (important) or 5 (very important). Furthermore, we tested whether the percentage of finance providers and farmers who perceived a requirement to be important was significantly different across finance provider types, using a chi square test.

We then used a gap analysis to compare farmer knowledge of the important finance requirements with the important requirements as stated by the finance providers. First, we selected the five requirements, which were rated as most important for each finance provider type. For each finance provider type, we then matched these five most important requirements with the important requirements identified by each farmer. A knowledge score of 1 indicates that a farmer correctly identified all five requirements as important. A knowledge score of 0.8 indicates that a farmer correctly identified four of the five requirements as important.

Lastly, we used a censored regression model to investigate the factors, which determine farmer knowledge of the importance of finance requirements. The generic form of a censored model [44] is:

$$y_{i}^{*}=x_{i}^{\prime }\beta +\varepsilon_{i},\;i=1,2,\ldots ,N$$ (1)

Following the generic form above, our model is:

$$y_{ij}^{*}=\beta_{1}+\beta_{2}age+\beta_{3}educ+\beta_{4}finexp_{j}+\beta_{5}fexp+\beta_{6}fsize+\varepsilon_{ij}$$ (2)

i=1,2,………,434(farmers).

j=1,2,………,5(finance provider types).

Where the dependent variable y_ij_* is the knowledge score, which captures the knowledge score of farmer *i* of the requirements set by finance provider *j*. The index *j* distinguishes bank (j = 1), MFI (*j = 2*), farmers’ association (*j = 3*), trader (*j = 4*) and agricultural input kiosk (*j = 5*). The independent variables (*x*_*i*_) are the demographic and socioeconomic factors, which we expected to influence farmer knowledge. The demographic variables are age (*age*) and education level (*educ*). The socioeconomic variables are finance experience from a specific finance provider (*finexp*_*j*_), i.e. whether a farmer has previously obtained finance from a specific provider, farming experience (*fexp*) and farm size (*fsize*). The variable *finexp*_*j*_ is a dummy variable, which takes the value of 1 if a farmer had previously obtained finance from finance provider *j*, and 0 otherwise. As before *j* takes the value 1, …, 5 to indicate experience with obtaining credit from a bank, MFI, farmers’ association, trader and agricultural input kiosk.

All variables, except dummy variables, were standardised by subtracting the variable-specific mean from each variable, and dividing the result by the standard deviation [45]. Standardisation was performed to ensure that all variables contributed evenly to a scale when items were added together. We tested for homoscedasticity using the Breusch-Pagan test. Furthermore, we checked for multicollinearity by calculating the Variance Inflation Factors (VIF) for each variable. Following the method of VIF of Rook et al. [46], results showed that the VIF of all variables were below 10, which implies that there was no multicollinearity problem in the model. We also tested for the presence of reverse causality in *finance experience* and *farm size*. For example, reverse causality in *farm size* could occur if having more knowledge of the finance requirements to obtain credit from a bank also allows farmers to attract more finance and rent more land.

## Results and discussion

### Important requirements to obtain finance: Perspective of finance providers

Table 2 shows that the most important requirements to obtain finance vary among finance provider types. For instance, banks perceive *character*, *capacity*, and *farmer ability* as most important. Contrary to findings from previous studies [4, 28, 47], we find that *collateral* is not the most important requirement to obtain finance from a bank. *Collateral* may be less important for banks in Indonesia because of the subsidised credit programme provided by the Indonesian government. For example, in the KUR programme, farmers can borrow money from banks without any collateral. MFI have different requirements from banks, although they both provide credit. For instance, in addition to *character*, MFI also perceive *spouse knowledge* as an important requirement to obtain finance (Table 2). This result is in line with a finding from previous research, i.e. that women in Indonesia have better skills in managing finance compared to men [48].

**Table 2:** Important finance requirements from the perspective of different finance provider types ^a^.

Finance requirement	Bank(n = 6)	MFI(n = 5)	Farmers’ association(n = 30)	Trader(n = 6)	Agricultural input kiosk(n = 13)
Collateral ^b^^,^^c^	83.3	40.0	53.8	33.3	7.7
Character ^b^^,^^d^	100.0	100.0	53.8	83.3	46.2
Capacity ^b^^,^^e^	100.0	80.0	92.3	83.3	30.8
Capital ^b^^,^^f^	66.7	60.0	61.5	16.7	0.0
Condition ^b^^,^^g^	83.3	0.0	15.4	33.3	7.7
Loan size ^b^	66.7	80.0	76.9	50.0	15.4
Farmer ability ^b^^,^^h^	100.0	60.0	92.3	100.0	15.4
Farm size ^b^	83.3	60.0	84.6	83.3	15.4
Spouse knowledge ^b^^,^^i^	66.7	100.0	61.5	0.0	7.7
Membership ^b^^,^^j^	33.3	60.0	100.0	66.7	15.4
Sales contract ^b^^,^^k^	50.0	20.0	53.9	100.0	15.4

^a^ Percentage of respondents who scored the factors as important or very important (score 4 and 5).

^b^ Indicates significant difference between finance provider types (P≤0.05).

^c^ *Collateral*: a farmer’s guarantee letters, such as land and vehicle certificates.

^d^ *Character*: a farmer’s history of loan repayments.

^e^ *Capacity*: the profitability of a farm.

^f^ *Capital*: savings.

^g^ *Condition*: the national political and macroeconomic situation in the country.

^h^ The ability to manage the farm.

^i^ Whether the spouse knows of the application for finance.

^j^ Membership of a registered farmers’ association.

^k^ Presence of a sales contract.

Source: Authors’ calculation

Providers of in-kind finance have different requirements. Farmers’ associations perceive *membership* as the most important requirement to obtain in-kind finance, whereas traders perceive this requirement to be less important. Instead, traders perceive *farmer ability* and *sales contract* to be the most important requirements (Table 2). This is consistent with Irianto et al. [49], who found that contracts between farmers and traders for the provision of credit and technical assistance, such as the terms of planting, can be implemented to overcome financial constraints.

Agricultural input kiosks perceive *character* as the most important requirement to obtain finance (Table 2). Similar results are shown by Schreiner [50], who also found that a person’s history of credit repayment is important for a lender to provide a loan. Furthermore, credit history, which represents the ability to repay debts, is important due to its relation to the probability of default in a credit programme [51].

Our results show that the requirements differ significantly between finance providers (P≤0.05), except for *character*, which is perceived as relatively important by all provider types (Table 2).

### Importance of finance requirements: Perception and knowledge of farmers

Farmers’ perceptions of the important requirements to obtain finance also vary across finance provider types (Table 3). From the perspective of farmers, the most important requirement to obtain finance from a farmer’s association is *membership*. However, only 80 percent of farmers perceive *membership* as very important (Table 3). Some farmers, especially farmers who are not a member of a registered farmers’ association, appear to be unaware that *membership* is very important to obtain finance from this provider. Farmers perceive *collateral* as the most important requirement to obtain finance from a bank, while banks do not perceive collateral as the most important requirement. This may be explained by the different requirements set for commercial and subsidised credit. Also, Deckiyanto found that banks did not provide their customers sufficiently detailed information on subsidised credit programmes [52]. Furthermore, farmers perceive *character* as important to obtain finance from a MFI, trader and agricultural input kiosk.

**Table 3:** Farmers’ perceptions of the important requirements to obtain finance per finance provider type ^a^ (n = 434).

Finance requirement	Bank	MFI	Farmers’ association	Trader	Agricultural input kiosk
Collateral ^b^^,^^c^	97	59	32	12	8
Character ^d^	80	70	58	51	53
Capacity ^b^^,^^e^	57	34	32	16	11
Capital ^b^^,^^f^	88	48	29	2	2
Condition ^b^^,^^g^	18	15	21	6	5
Loan size ^b^	73	58	46	32	25
Farmer ability ^b^^,^^h^	46	22	30	16	10
Farm size ^b^	34	20	23	13	10
Spouse knowledge ^b^^,^^i^	94	69	39	22	18
Membership ^b^^,^^j^	15	14	80	4	4
Sales contract ^b^^,^^k^	14	9	15	40	5

^a^ Percentage of farmers who scored the factors as important or very important (score 4 and 5).

^b^ Indicates significant difference between farmers’ perception to get finance from each finance provider type (P≤0.05).

^c^ *Collateral*: a farmer’s guarantee letters, such as land and vehicle certificates.

^d^ *Character*: a farmer’s history of loan repayments.

^e^ *Capacity*: the profitability of a farm.

^f^ *Capital*: savings.

^g^ *Condition*: the national political and macroeconomic situation in the country.

^h^ The ability to manage the farm.

^i^ Whether the spouse knows of the application for finance.

^j^ Membership of a registered farmers’ association.

^k^ Presence of a sales contract.

Source: Authors’ calculation.

A comparison of farmers’ and finance providers’ assessments shows that the requirements perceived as important by farmers often do not match the requirements set by the finance providers. Banks perceive *character*, *capacity* and *farmer ability* as the most important requirements (Table 2), whereas farmers perceive that banks do not focus on these requirements (Table 3). Farmers also perceive *farmer ability* and *sales contract* as not the most important to obtain finance from a trader (Table 3), whereas these variables are the most important requirements from the perspective of the traders (Table 2).

Our results show that farmers’ perceptions of the important requirements for obtaining finance are significantly different across finance provider types (P≤0.05). Farmers perceive that traders and input kiosks have the least strict requirements. The gap in the perceived importance of requirements between finance providers and farmers is illustrated by the relatively low knowledge scores of farmers, as shown in Table 4. These scores imply that the majority of farmers have a relatively little knowledge of the requirements to obtain finance from different finance provider types. Farmer knowledge is the highest with respect to the requirements of banks (0.63), whereas the lowest score is for knowledge of the requirements of the kiosks (0.17).

**Table 4:** Mean farmer knowledge scores and coefficients from the censored regression of knowledge scores on the influencing factors (n = 434), standard errors in parentheses.

Variable	Bank	MFI	Farmers’ association	Trader	Agricultural input kiosk
Farmer knowledge score	0.63	0.56	0.42	0.27	0.17
Constant	0.63(0.01)	0.53(0.02)	0.40(0.02)	0.22(0.02)	0.07(0.02)
Age (years)	-0.01(0.02)	0.02(0.02)	-0.03(0.02)	0.00(0.02)	0.03(0.02)
Education level (years)	-0.02(0.01)	-0.01(0.02)	0.05 ^a^(0.01)	-0.02(0.02)	-0.03 ^a^(0.01)
Finance experience ^c^ (dummy variable)	-0.03(0.03)	0.08(0.06)	0.05 ^b^(0.03)	0.03(0.04)	0.09 ^a^(0.03)
Farming experience (years)	-0.05 ^a^(0.02)	-0.08 ^a^(0.02)	0.03(0.02)	-0.03(0.02)	-0.08 ^a^(0.02)
Farm size (hectares)	0.02 ^b^(0.01)	0.00(0.02)	0.01(0.01)	0.03 ^a^(0.01)	0.04 ^a^(0.01)

^a^ Significant at 1%,

^b^ significant at 10% level.

^c^ Dummy variable representing whether the respondent had previously obtained finance from the finance provider type; 1 if yes, 0 otherwise.

Source: Authors’ calculation.

### Determinants of farmer knowledge of finance requirements

The results of the censored regression model to identify factors that influence farmer knowledge of the important finance requirements (referred to as farmer knowledge in this section) are presented in Table 4. Among the demographic factors, *education level* significantly and positively associates with farmer knowledge for farmers’ associations. This result implies that higher-educated farmers, *ceteris paribus*, have more knowledge of the finance requirements of farmers’ associations. However, we also found that *education level* has a significant, negative association with farmer knowledge for the agricultural input kiosk, which means that less-educated farmers have more knowledge of the important requirements to obtain finance from a kiosk. This result suggests that less-educated farmers might focus on obtaining finance from kiosks because they think they are unlikely to obtain finance from banks and MFI. Previous studies on access to finance did not explore the relation between knowledge of requirements and education level. A positive association between education and knowledge about loan-related questions was found by Cole et al. [53].

*Finance experience* is significantly associated with farmer knowledge for farmers’ associations. This implies that farmers who previously obtained finance from farmers’ associations are more knowledgeable about the requirements. Similarly, *finance experience* has a significant positive association with farmer knowledge for kiosks.

The other socioeconomic factors, i.e. *farming experience* and *farm size*, have a significant association with farmer knowledge for at least one type of finance provider. *Farming experience* has a significant, negative association with farmer knowledge for banks, MFI, and agricultural input kiosks, indicating that more-experienced farmers have less knowledge of the finance requirements of these three providers. More management experience may have made their farms more robust and therefore less dependent on access to finance. Previous studies on access to finance did not investigate the relation between knowledge of requirements and farming experience. Jabbour et al. [54] showed that farming experience was positively correlated with the knowledge of farmers, particularly in weed management.

Results in Table 4 also show that *farm size* has a significant, positive association with farmer knowledge for banks, traders and kiosks. This result implies that farmers with larger farms, *ceteris paribus*, have more knowledge of the finance requirements of these three providers, which suggests they focus on finding finance from sources that can improve their production, e.g. for adopting new technologies. Mauro and Mac Lachlan [38] showed that larger farmers focus on technology development, leading to improved knowledge of risk assessment on their farms.

We investigated the causality of the relation between several statistically significant variables and farmer knowledge, using a reverse causality test. For the farmers’ association, we found no evidence for reverse causality in the relation between farmer knowledge and *finance experience*. However, for agricultural input kiosks, the test provided evidence of reverse causality between farmer knowledge and *finance experience*. Hence, although the relation between *finance experience* from the kiosk and farmer knowledge is positive, the direction of the causality is unknown. Similarly, for banks and agricultural input kiosks, farmer knowledge has a significant, positive association with *farm size*. We found evidence for reverse causality between farmer knowledge and *farm size* for these two providers. Therefore, for banks and kiosks, the causality in the relation between *farm size* and farmer knowledge is also unknown.

The results of the paper cannot be directly compared to other regions or countries, except countries that have a similar farm and institutional environment as Indonesia. For instance, countries in South, Southeast and East Asia, and sub-Saharan Africa have comparable farm characteristics and our results may apply in these countries [55]. Nevertheless, there are no studies available on these countries with which our results can be compared.

## Conclusions and recommendations

This study compares farmer knowledge of the finance requirements of different finance provider types with the requirements as stated by the finance providers. Furthermore, it investigates the relation between demographic and socioeconomic factors and farmer knowledge of finance requirements. Data for the study were collected in Indonesia, from 43 finance providers and 434 horticultural farmers who cultivate mango, mangosteen, chili, and red onion.

Finance in Indonesia can be obtained from different finance provider types: banks, MFI, farmers’ associations, traders and agricultural input kiosks. Banks and MFI provide credit, whereas farmers’ associations and traders provide in-kind finance, and agricultural input kiosks provide flexible payment for inputs.

We find that the most important requirements to acquire finance vary among the finance provider types. Although banks and MFI both provide credit, they focus on different requirements. Banks perceive *character* in terms of the history of loan repayments, the *capacity* of farmers to pay back the loan, and *farmer ability* to manage their farms as very important requirements. MFI focus on *character* and *spouse knowledge* of the finance application. Furthermore, to obtain access to in-kind finance, farmers’ associations require farmers to have *membership* of a registered farmers’ association, whereas traders perceive *farmer ability* and presence of a *sales contract* as the most important requirements. Agricultural input kiosks perceive the *character* of the farmer to be the most important requirement.

Overall, farmers are found to have relatively little knowledge of the different requirements, which are important for each type of finance provider. The censored regression model shows that the factors influencing farmer knowledge of the requirements differ according to the type of finance provider. Farmer knowledge is positively associated with *finance experience*, especially for finance from farmers’ associations. *Education level* and *farming experience* have significant associations with farmer knowledge of the requirements of at least one type of finance provider.

The results of this study can be used to design strategies to improve farmer knowledge of finance and subsequent access to finance. Awareness campaigns could improve farmer access to finance by increasing farmer knowledge of the diversity of requirements among the finance provider types. Involving farmers who already have experience with different sources of finance likely increases the success of such campaigns.

## Supporting information

S1 Fig Conceptual framework. (TIFF)

S1 Table Data of finance providers. (XLSX)

S2 Table Data of farmers. (XLSX)

S1 File Questionnaire for finance providers. (DOCX)

S2 File Questionnaire for farmers. (DOCX)
