# Supplementary figures and images for: Access to finance from different finance provider types: Farmer knowledge of the requirements
Source: PLoS One. 2017 Sep 6;12(9):e0179285. doi: 10.1371/journal.pone.0179285 (PMC5587102; doi:10.1371/journal.pone.0179285)

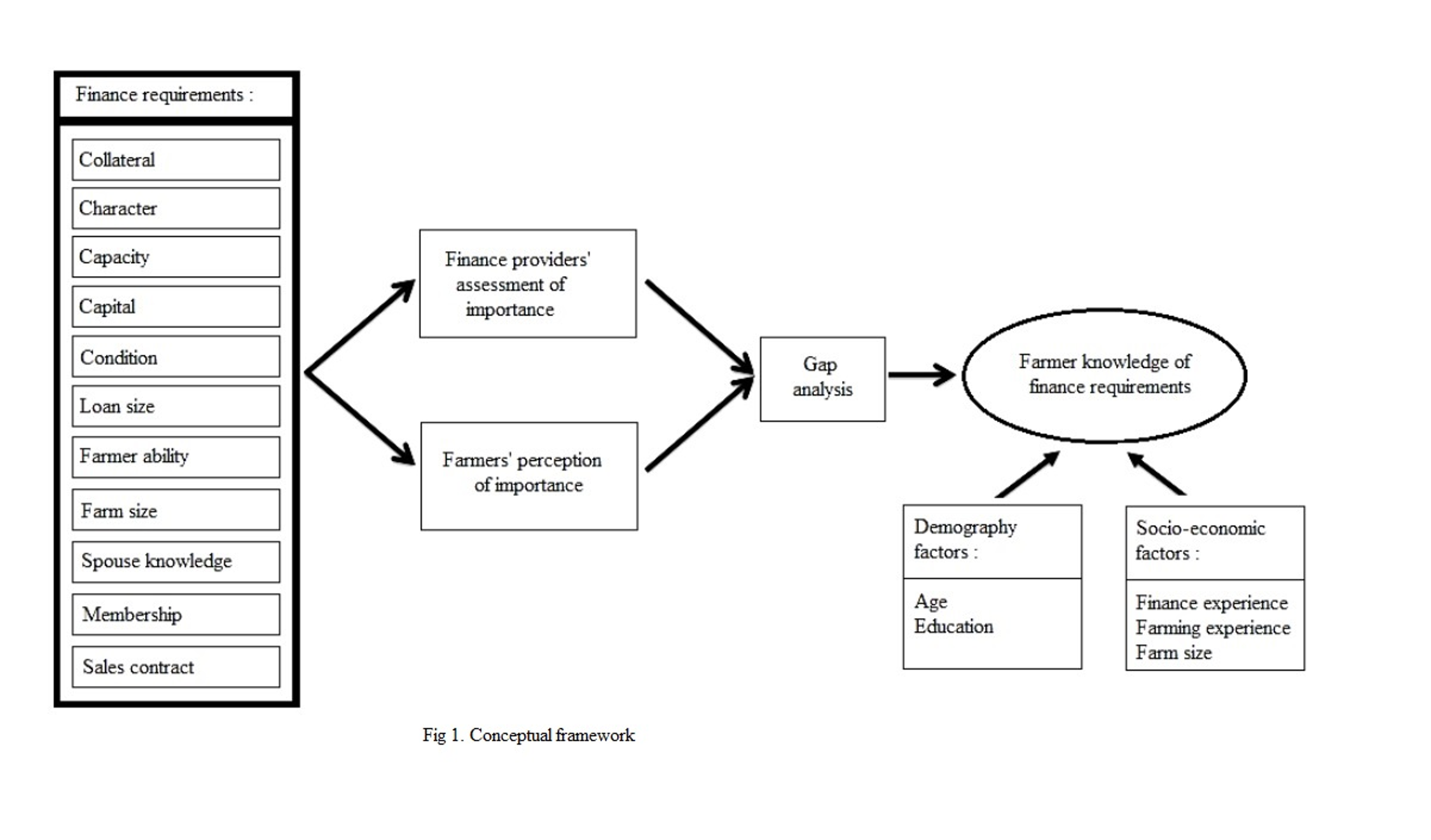

Supplement: S1 Fig — (TIFF) [file pone.0179285.s001.tiff]
