# Supplementary material for: Access to finance from different finance provider types: Farmer knowledge of the requirements
Source: PLoS One. 2017 Sep 6;12(9):e0179285. doi: 10.1371/journal.pone.0179285 (PMC5587102; doi:10.1371/journal.pone.0179285)
Supplement: S1 File — (DOCX) [file pone.0179285.s004.docx]

**S1 File. Questionnaire for finance providers**

**Access to finance from different finance providers: the requirements of finance provider**

1. Finance provider characteristics

| 1.1. Name of finance provider |  |
| --- | --- |
| 1.2. Address |  |
| 1.3. Number of employees (full time equivalent) |  |
| 1.4. Facilities |  |

1. What is the importance of the following requirements to access finance from your institution ?

| Requirement  Rating from 1 to 5 (1=not important at all; 2= relatively unimportant; 3= not important/neither unimportant; 4=relatively important; 5= very important | | | | | | | | | | |
| --- | --- | --- | --- | --- | --- | --- | --- | --- | --- | --- |
| Collateral ^a^ | Character ^b^ | Capacity ^c^ | Capital ^d^ | Condition ^e^ | Loan size | Farmer ability ^f^ | Farm size | Spouse knowledge ^g^ | Membership ^h^ | Sales contract ^i^ |
|  |  |  |  |  |  |  |  |  |  |  |
|  |  |  |  |  |  |  |  |  |  |  |
|  |  |  |  |  |  |  |  |  |  |  |
|  |  |  |  |  |  |  |  |  |  |  |
|  |  |  |  |  |  |  |  |  |  |  |
|  |  |  |  |  |  |  |  |  |  |  |
|  |  |  |  |  |  |  |  |  |  |  |
| ^a^ A farmer’s guarantee letters, such as land and vehicle certificates.  ^b^ A farmer’s history of loan repayments.  ^c^ The profitability of a farm.  ^d^ Savings.  ^e^ The national political and macroeconomic situation in the country.  ^f^ The ability to manage the farm.  ^g^ Whether the spouse knows of the application for finance.  ^h^ Membership of a registered farmers’ association.  ^i^ Presence of a sales contract. | | | | | | | | | | |

Thank you very much for your participation.
