# Supplementary material for: Access to finance from different finance provider types: Farmer knowledge of the requirements
Source: PLoS One. 2017 Sep 6;12(9):e0179285. doi: 10.1371/journal.pone.0179285 (PMC5587102; doi:10.1371/journal.pone.0179285)
Supplement: S2 File — (DOCX) [file pone.0179285.s005.docx]

**S2 File. Questionnaire for farmers**

**Access to finance from different finance providers: farmer knowledge of the requirements**

Farmer ID

| Name of Farmer |  |
| --- | --- |
| Address |  |
| Interviewer |  |

1. Farmer and farm characteristics

| 1.1. Age (years) |  |
| --- | --- |
| 1.2. Educational background (years) |  |
| 1.3. Experience in agricultural activities (years) |  |
| 1.4. Total farm size (hectares) |  |

1. Access to finance

| 2.1. Did you obtain one of the following sources of finance before 2013 (including 2013)? | Yes | No |
| --- | --- | --- |
| 1. Commercial credit from bank |  |  |
| b. Subsidised credit from bank |  |  |
| c. Commercial credit from microfinance institution |  |  |
| d. In-kind finance from farmers’ association |  |  |
| e. In-kind finance from trader |  |  |
| f. Flexible payment to agricultural input kiosk |  |  |
| g Finance from family/relatives/neighbour/friends |  |  |

2.2. What is the importance of the following requirements to access finance from various sources ?

| Sources of finance | Requirement  Rating from 1 to 5 (1=not important at all; 2= relatively unimportant; 3= not important/neither unimportant; 4=relatively important; 5= very important | | | | | | | | | | |
| --- | --- | --- | --- | --- | --- | --- | --- | --- | --- | --- | --- |
|  | Collateral ^a^ | Character ^b^ | Capacity ^c^ | Capital ^d^ | Condition ^e^ | Loan size | Farmer ability ^f^ | Farm size | Spouse knowledge ^g^ | Membership ^h^ | Sales contract ^i^ |
| Commercial credit from bank |  |  |  |  |  |  |  |  |  |  |  |
| Subsidised credit from bank |  |  |  |  |  |  |  |  |  |  |  |
| Commercial credit from microfinance institution |  |  |  |  |  |  |  |  |  |  |  |
| In-kind finance from farmers’ association |  |  |  |  |  |  |  |  |  |  |  |
| In-kind finance from trader |  |  |  |  |  |  |  |  |  |  |  |
| Flexible payment to agricultural input kiosk |  |  |  |  |  |  |  |  |  |  |  |
| Finance from family/relatives/neighbour/friends |  |  |  |  |  |  |  |  |  |  |  |
| ^a^ A farmer’s guarantee letters, such as land and vehicle certificates.  ^b^ A farmer’s history of loan repayments.  ^c^ The profitability of a farm.  ^d^ Savings.  ^e^ The national political and macroeconomic situation in the country.  ^f^ The ability to manage the farm.  ^g^ Whether the spouse knows of the application for finance.  ^h^ Membership of a registered farmers’ association.  ^i^ Presence of a sales contract. | | | | | | | | | | | |

Thank you very much for your participation.
